# Performance Assessment of Full-Scale Wastewater Treatment Plants Based on Seasonal Variability of Microbial Communities via High-Throughput Sequencing

**DOI:** 10.1371/journal.pone.0152998

**Published:** 2016-04-06

**Authors:** Tang Liu, Shufeng Liu, Maosheng Zheng, Qian Chen, Jinren Ni

**Affiliations:** 1 Department of Environmental Engineering, Peking University, Beijing, 100871, China; 2 Key Laboratory of Water and Sediment Sciences, Ministry of Education, Beijing, 100871, China; NERC Centre for Ecology & Hydrology, UNITED KINGDOM

## Abstract

Microbial communities of activated sludge (AS) play a key role in the performance of wastewater treatment processes. However, seasonal variability of microbial population in varying AS-based processes has been poorly correlated with operation of full-scale wastewater treatment systems (WWTSs). In this paper, significant seasonal variability of AS microbial communities in eight WWTSs located in the city of Guangzhou were revealed in terms of 16S rRNA-based Miseq sequencing. Furthermore, variation redundancy analysis (RDA) demonstrated that the microbial community compositions closely correlated with WWTS operation parameters such as temperature, BOD, NH_4_^+^-N and TN. Consequently, support vector regression models which reasonably predicted effluent BOD, SS and TN in WWTSs were established based on microbial community compositions. This work provided an alternative tool for rapid assessment on performance of full-scale wastewater treatment plants.

## Introduction

Activated sludge (AS) process has been most widely used to treat domestic sewage for a century due to its high treatment efficiency and low cost. Biological wastewater treatment, as the largest application area of biotechnology, accelerates the beneficial activities of naturally occurring microorganisms, removing organic pollutants and nutrients via metabolism [1]. Thus, microbial communities in AS ecosystems are crucial for well-performing bioreactors. However, maintaining municipal wastewater treatment systems (WWTSs) is still based on empirical relationships between physicochemical and operational parameters and reactor performance, which is not reliable enough for stable performance [2]. A systematic understanding of bacterial communities as a function of environmental factors and how they influence the performance is vital to improve process performance stability and provide important guidance in diagnosis and prognosis.

In recent years, the microbial communities of AS in full-scale and lab-scale bioreactors were shown to be highly diverse and variable. Numerous studies were conducted to investigate AS microbial community compositions varied across bioreactors and time. A wide range of discernible temporal patterns of AS microbial communities were proposed, particularly within specific microbial subpopulations such as nitrifiers [3], denitrifiers [4] and phosphorus-accumulating organisms [5]. In a full-scale WWTS, Kim et al. [6] found the significantly different temporal patterns between the rare taxa and the general taxa of AS, in which the rare taxa showed a higher diversity and a more fluctuating pattern than the general taxa. Variability of AS microbial communities across different bioreactors was investigated at scales ranged from single wastewater treatment plant to globally distributed wastewater treatment plants. For single wastewater treatment plant which had two disparate treatment systems operating in parallel, distinction between the microbial community compositions of the two systems was detected [7]. Clear geographical disparity was also showed among the AS samples from Asia and North America [8]. Other studies showed that the variance of bacterial communities explained by geographic location is smaller than other factors in 14 WWTSs located in 4 cities in China [1]. This also implied that variability of AS microbial communities across bioreactors and time might be worthy of more attention in wastewater treatment processes.

Biological treatment is an extremely complex system with deeply diverse microbial communities and exhibits highly nonlinear characteristics [9,10]. To predict the performance of bioreactor, artificial intelligence approaches, such as artificial neural networks, adaptive neuro fuzzy-fuzzy interference system and fuzzy logic, have proved to be useful tools due to their high accuracy in dealing with complicated systems [9–11]. As a kind of data-based machine learning model, support vector regression (SVR) model is a method with the pattern of supervised learning, and based on statistical theory, VC dimension theory and structural risk minimum principle. SVR model exhibits many unique advantages in solving small-sample, nonlinear and high-dimensional recognizing problems [12]. It has been used to predict the removal efficiency of settling basins and the results are found to be better than the neural network approach [11]. Additionally, SVR model also shows a higher prediction accuracy in the training stage and the validation stage to predict effluent concentration in a full-scale WWTS [13]. However, most models predict the effluent quality in terms of environmental factors rather than microbial community information which may have greater influence on the performance of wastewater treatment. Recently, high-throughput sequencing has been widely applied for characterizing AS microbial community compositions both in lab-scale systems and full-scale plants [8], which could obtain more precise inventories of microorganisms.

In this study, AS samples were quarterly collected from eight full-scale WWTSs in Guangzhou, China from July 2013 to April 2014. 16S rRNA-based Miseq sequencing was used to characterize microbial communities of AS samples. The aim of this study was to seek an alternative for rapid assessment on performance of full-scale wastewater treatments facilities via seasonal variability of AS microbial community structures with the help of high-throughput sequencing.

## Materials and Methods

### WWTSs description and sampling

AS samples were taken from the aeration tanks of eight WWTSs (S1 Table) in Guangzhou, China. Names of these WWTSs were designated as random letters as part of the sample confidentiality agreement. These WWTSs treat mainly municipal wastewater except S1 with twenty percent influent of industrial wastewater.

AS samples were collected quarterly from these eight systems from June 2013 to April 2014 with water temperature ranged from 15 to 30°C. Samples were taken in June, October, January and April, representing summer, fall, winter and spring, respectively. For archiving, 50 ml sludge sample was taken in each system, and immediately placed in an ice box and transferred to the laboratory for DNA extraction. Sampling time, influents, effluent and operational parameters for the eight systems are listed in S2 Table.

### DNA extraction, PCR amplification and MiSeq sequencing

The samples from L4 taken in June 2013 and D1 in January 2014 were divided into two aliquots named as L4-Summer1, L4-Summer2, D1-Winter1 and D1-Winter2, respectively. The duplicate samples were treated as independent samples to evaluate the reproducibility of the methods applied. Total genomic DNA was isolated from each sample in duplicate using the FastDNA^®^ SPIN Kit for Soil (MP Biotechnology, Solon, OH, USA) that was regarded as superior to many others [14] according to the manufacturer's protocol.

DNA was PCR-amplified using barcoded primers targeting bacterial V4 region of 16S rRNA genes. Primer pair 515F (5’-GTGCCAGCMGCCGCGGTAA-3’)—806R (5’-GGACTACHVGGGTWTCTAAT-3’) was selected due to accurate taxonomic information and few biases for various bacterial taxa [15]. These PCR products of the V4 region of 16S rRNA genes were mixed in equimolar amounts and paired-end sequenced (2×150) using the Illumina MiSeq platform according to the manufacturer's instructions. All 16S rRNA sequences from Miseq sequencing have been deposited into the NCBI short-reads archive database with accession number SRR2153416-SRR2153448.

### Sequence processing and statistical analysis

The raw sequencing data from Miseq sequencing was processed by QIIME v.1.7.0 [16] and UPARSE pipeline [17]. Sequences either containing Ns, or with lengths shorter than 200 bp, or average quality score less than 25, were filtered. Sequences were de-replicated and singleton was removed from consideration. The remaining sequences were clustered into operational taxonomic units (OTUs) at 97% pairwise identity using UPARSE [17]. Chimera was checked against a reference Gold database (http://drive5.com/uchime/gold.fa) [17] by UCHIME. The representative sequence for each OTU was aligned to the Greengenes database (version 13.5) of high-quality sequences with the default confidence threshold (0.5) [18]. All the filtered reads including singletons and duplicate sequences were mapped back to the OTUs.

Alpha-diversity indices, including Shannon, Simpson, Chao1 and PD whole tree, were calculated by QIIME 1.7.0 for each sample. Relative abundance of OTUs was calculated in each sample, and then was used to calculate pairwise similarities among samples using the Bray-Curtis similarity coefficient. The cluster analysis was conducted to group the microbial communities based on the Bray-Curtis similarity coefficient and was visualized using principal coordinates analysis (PCoA). Analysis of Similarity Statistics (ANOSIM) was calculated to test the significance of differences among a priori grouping strategy based on the result of PCoA. Redundancy analysis (RDA) was used to assess the contribution of environmental variances to the variances of bacterial communities. Function envfit within the R package vegan which fits environmental vectors onto ordination was applied to assess the influence of environmental variables on the microbial community structures [19]. Similarity matrices, PCoA, ANOSIM and RDA were carried out using R (3.1.1).

### SVR models construction

Libsvm package [20] with R (3.1.1) was used for building SVR models to predict effluent BOD, SS, NH_4_^+^-N, TN and TP. The total 33 samples were divided into two different groups: 27 as training data set and 6 remaining as validation data set. The OTUs data was chosen as the model input data. The OTUs data had more than 5000 variables, so principal component analysis (PCA) was applied to reduce dimensionality firstly. The effluent data was normalized to range from 0 to 1. After that, the dimensions reduced data was used as input data to train the SVR models, to obtain the best model parameters and make the model have the ability to predict reactor process performance. The optimal parameters of the model were determined by grid search method, that all the possible combinations of parameter values are evaluated and the best combination is retained. After determining the model parameters, the effluent BOD, SS, NH_4_^+^-N, TN and TP concentration of 6 validation samples would be predicted by the SVR model, the values were compared to the measured values to evaluate the prediction accuracy. Robust estimation of model was tested by changing the training dataset and validation dataset for 5 times. Sensitivity analysis was performed to quantify the relative importance of each input to the effluent prediction by One-factor-at-a-time. Briefly, one input varied ±10%, others kept invariant, then the dataset was applied to these models to assess the effect of the input on effluent prediction. The other inputs were repeated in the same way.

## Results and Discussion

### Compositions of microbial communities in AS

High-throughput sequencing technology offers aid to uncover greatly diverse microbial communities. After filtering the low quality sequences, a total of 2,197,507 effective sequences were yielded for the 33 samples, and clustered to 5,409 OTUs with 3% of nucleotide cutoff. Individual sample contained much smaller number of OTUs from 715 to 1003. The multiple alpha-diversity indices are shown in S3 Table.

### Taxa distribution at the phylum level

**Fig 1:**
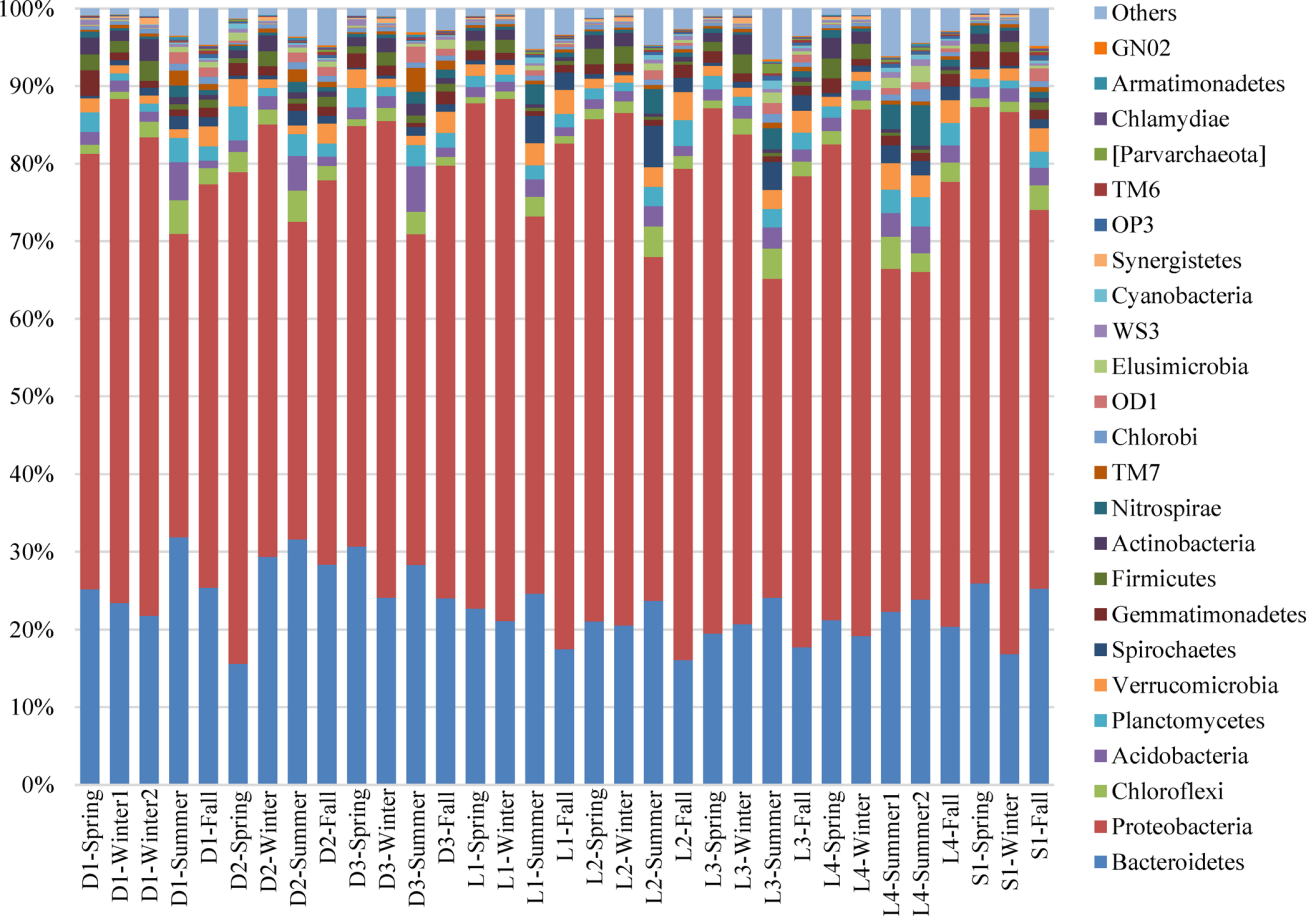
Abundances of different phyla in the 33 activated sludge (AS) samples. The abundance is presented in terms of percentage in total effective bacterial sequences in a sample.

A total of 58 phyla were detected in the 33 samples from 8 WWTSs. As shown in Fig 1, *Proteobacteria* is the most abundant phylum in all samples, accounting for 38–70% (average 56.3%) of total effective bacterial sequences, followed by *Bacteroidetes* corresponding to 15–32% (average 23.0%). These two phyla represented approximately 65–88%. Within *Proteobacteria*, *β-Proteobacteria* was the predominant group (33–54%), followed by *δ-Proteobacteria* (22–41%), *γ-Proteobacteria* (9–26%) and α-*Proteobacteria* (3–10%). Within the *β-Proteobacteria*, twelve orders were identified. *Rhodocyclales* was the dominant group within a range of 32–95% (average 52%), followed by *Burkholderiales*, *Neisseriales* and *Thiobacterales*, representing 20–49%, 1–24% and 1–11%. Within *Bacteroidetes*, all samples showed a similar composition at the class level, with *Saprospirae* as the major subdivision, followed by *Sphingobacteriia*, *Flavobacteria*, *Bacteroidia*, and *Cytophagia*. The subdominant phyla (average abundance >1%) included *Chloroflexi* (2.0%), *Acidobacteria* (2.0%), *Planctomycetes* (2.0%), *Verrucomicrobia* (2.0%), *Spirochaetes* (1.3%), *Gemmatimonadetes* (1.3%), *Firmicutes* (1.2%), *Actinobacteria* (1.2%) and *Nitrospirae* (1.1%), which was also considered as the important part in AS.

The microbial community compositions of the eight WWTSs showed typical AS communities [7,8]. Among all samples, *Proteobacteria* is the most abundant phylum. It is similar to the analytical results of bacterial communities in AS ecosystems from 14 sewage wastewater treatment plants, located in Canada, USA, Singapore, and China using 454 pyrosequencing [8]. Within *Proteobacteria*, in this study, the *β*-*Proteobacteria* is the dominant for most samples, which was consistent with most WWTSs based on other reports [1,8]. However, it is different from some studies using microarray [21], which showed that *α-Proteobacteria* was the most abundant subdivision. *Bacteroidetes*, *Actinobacteria*, and *Firmicutes* also played important role in microbial communities of 33 samples in this study, which is similar to a few previous studies on AS using microarray [21] and Pyrosequencing [8].

**Fig 2:**
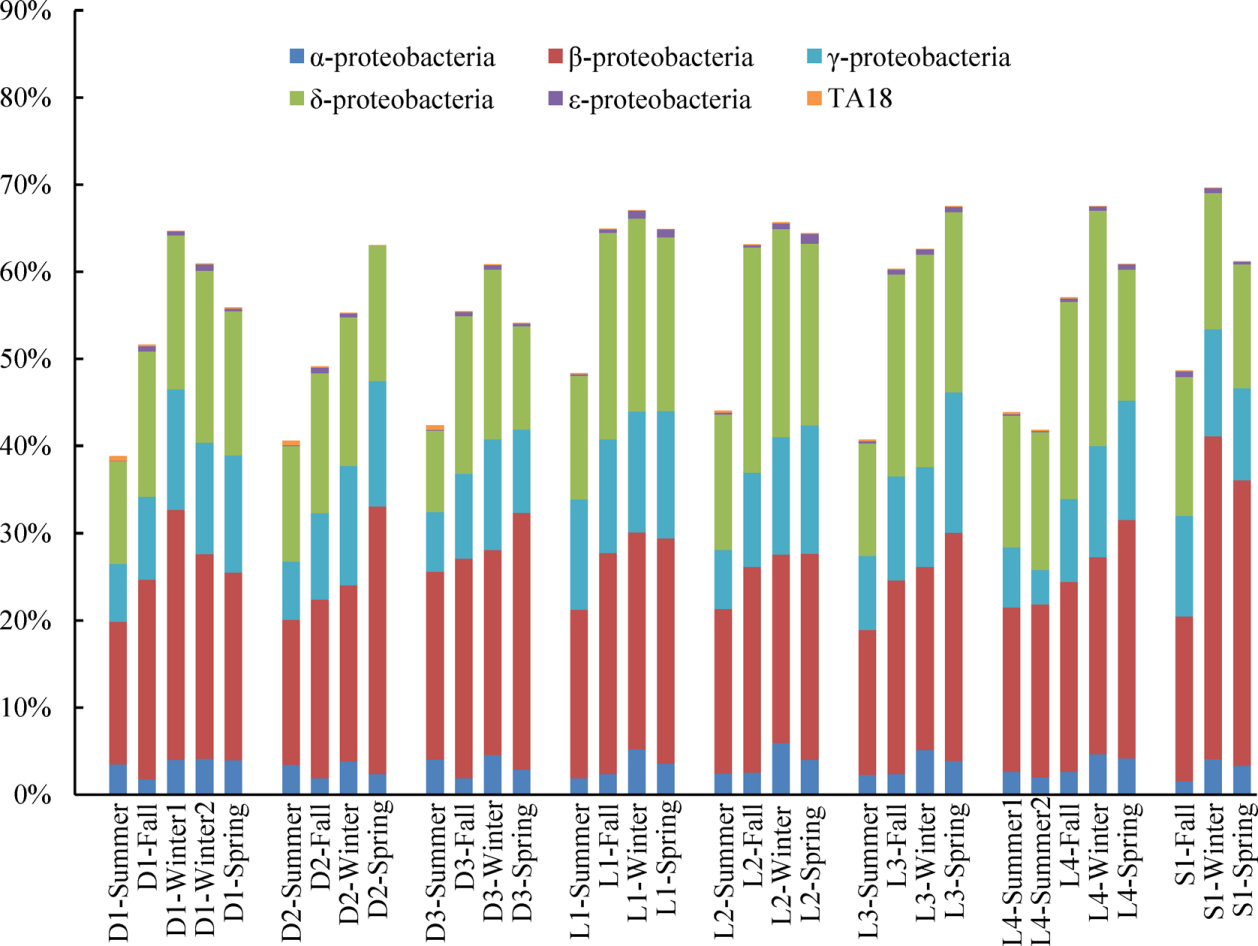
The seasonal variation of abundances of *Proteobacteria*. The abundance is presented in terms of percentage in total effective bacterial sequences in each sample.

*Proteobacteria*, as the most important phylum in all samples, varied with seasons. Fig 2 shows the seasonal variation of *Proteobacteria* in the 33 AS samples clearly. The lowest content (39.5–48.8%) took place in summer samples for all systems. The highest content (56–70%) occurred in winter samples, except for D2 and L3. For the *Proteobacteria*, the highest average content occurred in winter samples, which might be caused by some subdivisions of *Proteobacteria* belonged to cold-adapted microorganisms which exhibited rich diversity in cold environment, and formed a set of cold resistant mechanisms [22].

### Core and shared genera in all samples

At the genus level, 303 genera (S4 Table) have been identified in this study, in which 51 genera were shared by all 33 AS samples, accounting for 83% of the classified sequences, which showed a core microbial community across the 33 AS samples. Among all the samples, *Dechloromonas* was the most dominant genus, accounting for 21% on average of classified sequences, followed by *Zoogloea* (average content >10%). The other top 10 genera on average included *Nitrospira*, *Turneriella*, *Candidatus Accumulibacter*, *Sulfuritalea*, *Prosthecobacter*, *Nannocystis*, *Bdellovibrio* and *Sterolibacterium*, which usually occurred in WWTSs [8,23–25].

Core genera are thought to be the crucial part associated with the wastewater treating performance. In this study, *Dechloromonas* and *Zoogloea* are the dominant genera in all samples, which is similar to the results of AS samples from 14 sewage wastewater treatment plants all around the world [8]. *Dechloromonas* is frequently found in wastewater treatment plants, which was reported to be capable of anaerobic benzene degradation, denitrification and phosphorous removal [26,27]. *Zoogloea* is the typical AS bacterium that plays an important part in wastewater treatment by its ability to lower biological oxygen demand and by being the main agent for the flocculation of AS [28]. *Zoogloea ramigera* is known to form characteristic cell aggregates embedded inextracellular gelatinous matrices [29]. *Sulfuritalea* [23], *Nitrospra* [30] and *Candidatus Accumulibacter* [31] which are also abundant in this study have been proved to promote nitrogen and phosphorus removal in treating wastewater. Although some other abundant genera, such as *Turneriella*, *Prosthecobacter* and *Nannocystis* are commonly found to occur in WWTSs, the information about their existence and roles is limited. The high diversity of microbial communities shown in AS does not mean that all sequences can be classified. In fact, the short length of the 16S rRNA gene amplicons may influence the taxonomic classification accuracy, and this limitation for high-throughput sequencing technique need further modification.

### Variability of microbial community compositions across multiple seasons and WWTSs

**Fig 3:**
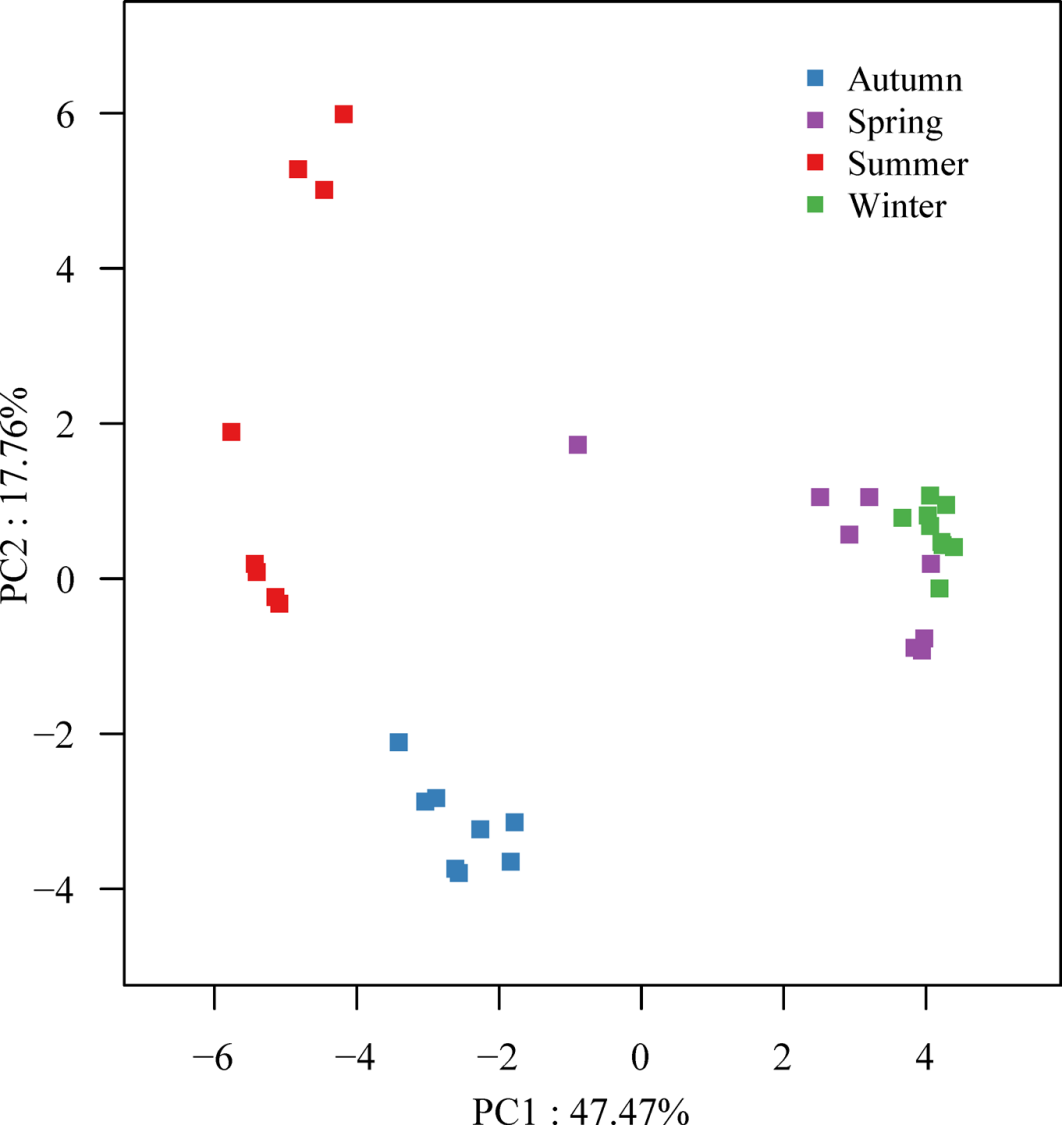
Principal coordinates analysis (PCoA) of 33 activated sludge (AS) samples by Bray-Curtis similarity coefficient. It was measured via 3% cutoff OTUs information. The dots were signed with different colors according to sampling seasons.

OTU-based Bray-Curtis similarity coefficients for beta-diversity of the bacterial communities are given in S5 Table. PCoA was conducted to evaluate similarities of all AS samples. Seasonal variation in communities based sampling time is shown in Fig 3. Four groups were distinguished in the first two dimensions: summer, autumn, winter and spring. The PC1 is clearly related to water temperature and explains 47.37% of the variation. The first two PCs totally explains 65.12% of the variation among the 33 samples. The samples in summer group seem more dynamic than other samples considering their relative wide distribution in the ordination, while good similarity on microbial communities among the samples in winter group is well characterized with their closer distribution. ANOSIM was also conducted to test the hypothesis that within-season microbial community similarities were greater than among-season similarities (Table 1). Global ANOSIM illustrated strong and significant variation in microbial communities across seasons (R = 0.72, p = 0.001). Moreover, six pairwise ANOSIM tests demonstrated significantly higher within-group similarity than between-group similarity at the p = 0.01 level.

**Table 1:** Analysis of Similarity Statistics (ANOSIM) test for significant differences between quarterly groupings in AS overall microbial community compositions. A priori grouping strategy was based on the result of principal coordinates analysis (PCoA).

Samples in different seasons	Significant difference	P-value
Global, with seasons nested	0.72	0.001
Summer vs. Fall	0.66	0.001
Summer vs. Winter	0.98	0.001
Summer vs. Spring	0.93	0.001
Fall vs. Winter	1	0.001
Fall vs. Spring	0.87	0.001
Winter vs. Spring	0.33	0.001

Previous studies of the variability and diversity of microbial communities in AS ecosystems are restricted to single dimensions, focusing on different bioreactors [8] or long-term time series [6] across environmental factors. Although Hai et al. [32] showed the bacterial community dynamics within a lab-scale and a full-scale bioreactor, the variability of the AS bacterial community compositions across bioreactors and time was not given. Therefore, we present a dataset containing varying microbial community compositions of AS, which could reflect both seasonal and locational influences on the performance of the 8 concerned WWTSs. The PCoA and ANOSIM based on OTUs allow further studies on patterns of microbial communities in 4 distinct groups across season variability. For the same season, AS samples taken from different WWTSs shared great similarity on microbial communities. However, those taken from the same WWTS demonstrated seasonal variations of the communities in spite of their same origin of inoculum and temporary continuity. Furthermore, higher temperature stress in winter (15–21°C) might lead to lower microbial diversity and less dynamic than in summer (29–30°C). Fortunato et al. [33] found that spatial variability overwhelms seasonal patterns in bacterioplankton communities across coastal margin, while different environments such as river, estuaries, plume and ocean were considered. Valentín-Vargas et al. [34] studied activated sludge microbial communities during a one-year period from two conventional activated sludge (CAS) bioreactors by T-RFLP. The results showed microbial community structures from the same bioreactors were more similar than those between different bioreactors. In our study, the WWTSs are located in the same city, which implies similar climatic and geographical environments and thereby seasonal patterns of AS microbial communities are expected to be overwhelmed location variability.

### Microbial community dynamics altered by environmental factors

**Fig 4:**
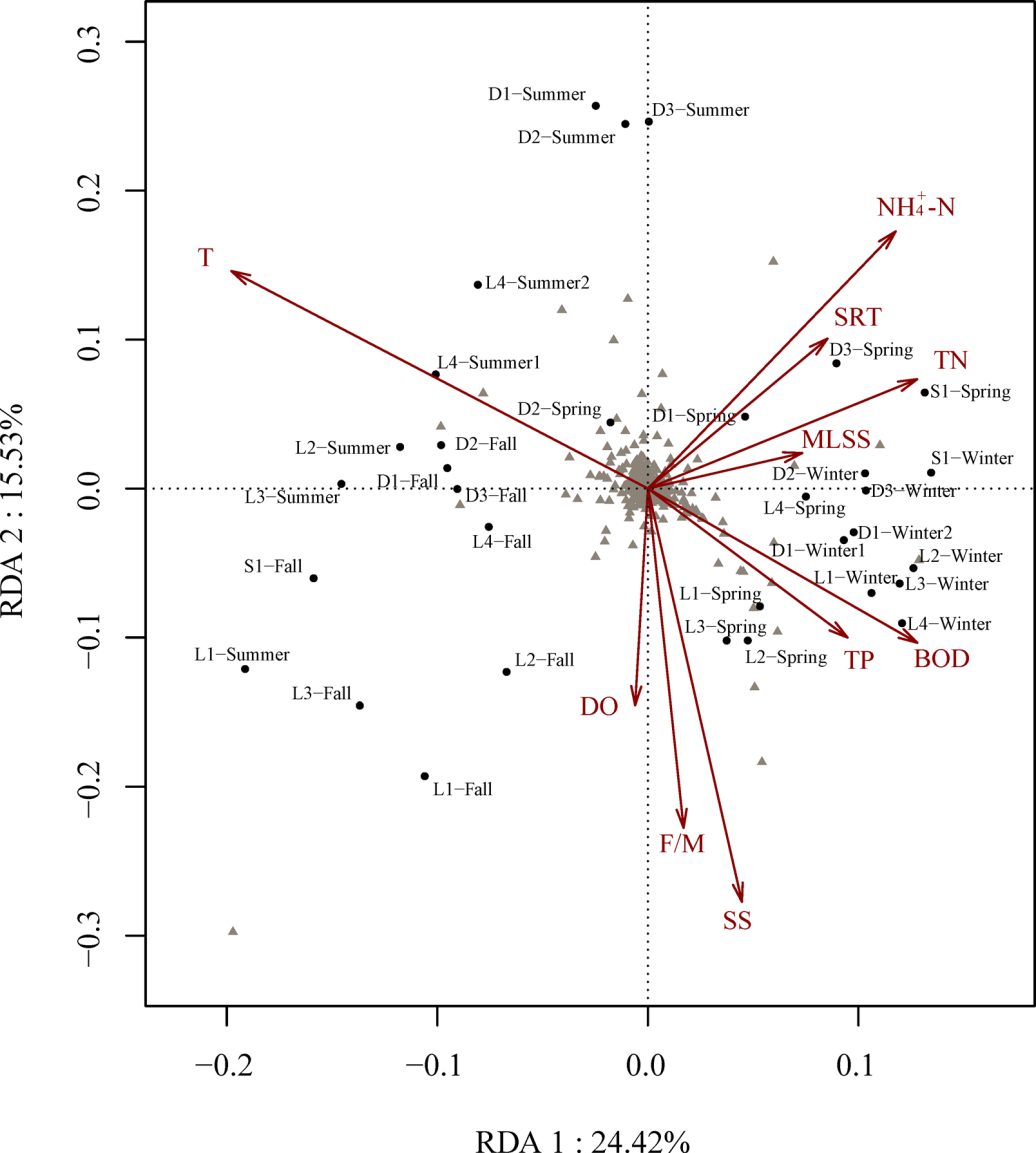
Redundancy analysis (RDA) of OTUs data and measurable variables in all samples. The arrows represent environmental parameters. The length of the arrows indicates the strength of the correlations and the angle of the arrows indicates the direction of variable increment. The triangles represent the species. The dots represent the samples.

The direct gradient ordination method RDA was performed to discern the possible influence of physicochemical and operational parameters on microbial community compositions (Fig 4). It explained the majority of variance in the species-environment correlations (62.7%). Based on Function envfit with 999 permutations, of 10 input explanatory variables, 5 were identified as significantly linked to microbial community variability at the p < 0.05 level. The length of an environmental variable arrow indicates the strength of the relationship between that variable and microbial community compositions. As such, temperature is the most important environmental parameter (r = 0.66, p = 0.001) dramatically affecting the microbial community compositions. The dominant taxa OTU1 and OTU9 increased across the temperature. Influent SS, BOD, NH_4_^+^-N and TN appears to strongly influence the compositions of microbial communities. OTU6 as the most abundant OTU showed decline with increasing NH_4_^+^-N.

Seasonal or locational variations in microbial communities might be influenced by many environmental factors, particularly on the rate of growth of individual taxa and physical parameters preventing different communities from interacting [35]. A deeper analysis of the compositions and dynamics of bioreactor microbial communities as a function of environmental factors is of great help to enhance treatment performance and management. Therefore, RDA ordination analysis indicated that temperature, a well-recognized variable in biological WWTSs, was most strongly and significantly associated with microbial community dynamics. The influence of temperature on microbial community compositions of AS across space or time has been noted previously. Spatial pattern was studied via 14 wastewater treatment plants in China, the canonical correspondence analysis results showed that the microbial community variance correlated most strongly with water temperature [1]. Based on the survey of temporal dynamic patterns of bacteria communities both in lab-scale and full-scale reactors via 454 pyrosequencing of 16S rRNA genes, the microbial community variance was significantly associated with water temperature [32]. Similar results have also been obtained in a lab-scale reactors treating industrial wastewater [36] and a full-scale wastewater treatment plant [3]. The observed correlation to temperature may be a reflection of seasonal periodicity in microbial community compositions. Furthermore, BOD plays a key role in shaping the microbial communities in WWTSs since organic loading is important carbon or energy source to heterotrophic microorganisms [6]. High organic loading could influence microbial diversity by reducing competition between the heterotrophic microorganisms [37]. In a lab-scale bioreactor under continuous steady-state conditions, Van Der Gast et al. [38] have also demonstrated that organic loading was an important structuring factor for the pattern of microbial community compositions and diversity via the denaturing gradient gel electrophoresis (DGGE) analysis. The influent concentration of TN and NH_4_^+^-N was also significantly linked to microbial community compositions. Nitrogen source would affect the growth of microorganisms and functional microbial communities relevant to nitrogen cycle. Previous studies indicated that ammonia concentration affects nitrification, nitrite accumulation and nitrifying microbial communities [39]. However, more than 30% of the community variance cannot be explained by the 10 environmental factors (main process operating parameters and influent parameters). It is reasonable to expect that some additional factors, such as stochastic factors [38], taxonomic relatedness and competition [40], organic toxicity of influent [41] and unmonitored factors (e.g. conductivity, oxidation reduction potential, etc.) [34], shape bacterial assembly in AS.

### Performance assessment using SVR model

**Fig 5:**
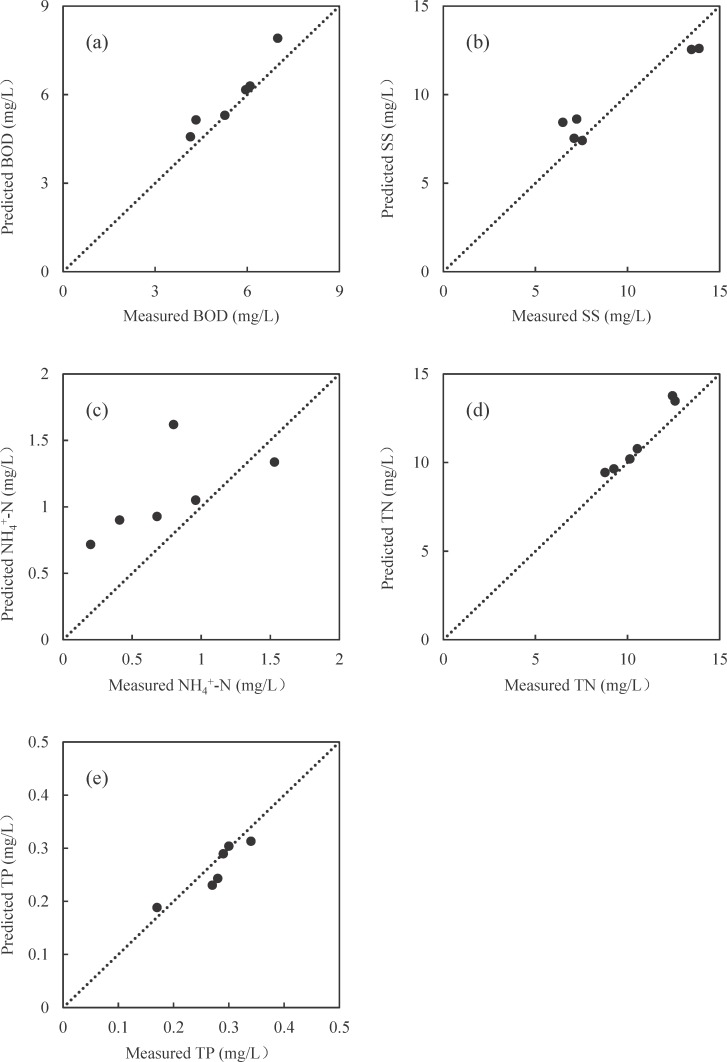
**Plot of the modeled versus measured BOD (a), SS (b), NH**_**4**_^**+**^**-N (c), TN (d), and TP (e) concentration in effluent from the validation tests.** The dashed line on each plot fits for the ideal “y = x” line which means the model perfectly fits the data set.

Microbial community compositions in the complex data set consists of 5412 OTUs in the present study. Thus, it was necessary to do dimensionality reduction before building SVR model, and 22 new variables were acquired. Furthermore, 27 AS samples were randomly selected for the training of SVR model, while the remaining 6 samples were used for model validation. The training results were listed in Table 2, which showed a very good training for BOD, SS and TN prediction models with the mean square error (mse) less than 0.008 and high correlation coefficients (r^2^) in the training sets, but poorer training for NH_4_^+^-N and TP with larger mse and smaller r^2^. Consequently, better validation was found for BOD, SS and TN models. Fig 5 showed reasonable agreement between the predicted and measured BOD, SS and TN with the r^2^ > 0.9. For NH_4_^+^-N, low correlation between the predicted and measured values was illustrated with r^2^ < 0.5. Robust estimation was tested by changing the training dataset and validation dataset (S6 Table). For BOD, SS and TN prediction models, r^2^ was > 0.8 in each permutation. The sensitivity ranking for the performance of the top 10 inputs to the effluent in BOD, SS and TN prediction models was made and the relevant results were summarized in Table 3. In BOD prediction model, OTU1 and OTU6 were the most sensitive inputs with variation range greater than 0.5%. In SS prediction model, OTU6 is of the first important input, followed by other 9 OTUs with variation range greater than 1%. For TN prediction model, the primary inputs were found with the order of OTU1, OTU6, OTU12 and OTU4. The taxonomy assignment of these OTUs was listed in S7 Table.

**Table 2:** Optimized support vector regression (SVR) models for effluent prediction in terms of BOD, SS, NH_4_^+^-N, TN and TP (all with microbial community compositions as inputs).

Water constituents	CVmse^a^	c^b^	g^c^	Training sets		Validation sets	
				mse	r^2^	mse	r^2^
BOD	0.0424	2.143	48.50	0.00652	0.935	0.00787	0.907
SS	0.0373	59.714	8.00	0.00771	0.931	0.01401	0.930
NH_4_^+^-N	0.0642	2.828	45.25	0.03196	0.717	0.03851	0.412
TN	0.0424	2.462	90.51	0.00700	0.942	0.00478	0.966
TP	0.0536	4.925	128.00	0.01201	0.848	0.02475	0.824

^a^CVmse: cross validation mse.

^b^c: the optimized regularization cost parameter

^c^g: the optimized kernel-specific parameter.

**Table 3:** Sensitivity rank of top 10 input variables in support vector regression (SVR).

BOD		SS		TN	
Input	Variation range	Input	Variation range	Input	Variation range
OTU1	0.78%	OTU6	2.54%	OTU1	1.77%
OTU6	0.56%	OTU3	2.03%	OTU6	0.89%
OTU12	0.32%	OTU4	1.81%	OTU12	0.71%
OTU7	0.21%	OTU1	1.06%	OTU4	0.52%
OTU9	0.11%	OTU7	0.92%	OTU10	0.22%
OTU17	0.08%	OTU5	0.86%	OTU5	0.18%
OTU11	0.07%	OTU14	0.81%	OTU14	0.17%
OTU13	0.06%	OTU2	0.72%	OTU3	0.16%
OTU5	0.06%	OTU10	0.55%	OTU20	0.16%
OTU3907	0.05%	OTU9	0.55%	OTU18	0.15%

Oxidation of ammonium to nitrite is conducted by ammonia oxidizing bacteria including only a few special genera, such as *Nitrosomonas*, *Nitrosospira* and *Nitrosococcus* [42]. Polyphospate accumulating organisms (PAOs) are responsible for the removal of phosphorus from wastewater and the most important PAOs have been identified as *Candidatus Accumulibacter phosphatis* [43] and *Tetrasphaera* [44]. These may be the reasons for the poorer prediction for NH_4_^+^-N and TP using microbial community compositions [45]. The better fit of predicted and measured BOD, SS and TN could be attributed to wide distribution of organic degrading bacteria and denitrifying bacteria. Similar results were derived by changing the training and validation datasets, which demonstrated the robustness of the models. Sensitivity analysis on the influence of microbial communities on system performance showed the lowest sensitive to the top 10 OTUs in BOD prediction model. Seshan et al. [45] showed that richness was more prominent in predicting effluent BOD in SVR model, and functional redundancy led by high richness might result in the low sensitivity of the OTUs. Such real-time models have been established using diversity indices of microbial communities for predicting reactor performance in a controlled experimental setting [45], but rarely done using community compositions for predicting effluent water quality in full-scale systems. With the SVR models, the performance of the full-scale bioreactors could be well assessed based on the core microbial community compositions.

## Conclusions

Our results demonstrated significant seasonal variability of microbial communities in eight full-scale WWTSs in Guangzhou, China. Based on the input information on microbial community compositions derived from high-throughput sequencing, the trained support vector regression models could reasonably predict for effluent BOD, SS and TN despite less satisfactory for NH_4_^+^-N and TP. This provided an alternative option for efficient assessment on their performance of full-scale wastewater treatment plants.

## Supporting Information

S1 Table List of 8 wastewater treatment systems. (XLSX)

S2 Table Characteristics of 8 wastewater treatment systems during sampling time. (XLSX)

S3 Table Different α-diversity indices of 33 samples. (XLSX)

S4 Table Abundance of genera across the 33 samples. (XLSX)

S5 Table Bray-Curtis similarity coefficients of microbial communities. (XLSX)

S6 Table Robust tested by changing the training dataset and validation dataset. (XLSX)

S7 Table The taxonomy assignment of the OTUs. (XLSX)
